# The Thyroid Condition and Residual Clinical Signs in 31 Existing Endemic Neurological Cretins After 42 Years of Iodine Supplementation in China

**DOI:** 10.3389/fendo.2022.911487

**Published:** 2022-07-08

**Authors:** Jianshuang Li, Yanhong He, Bingxuan Ren, Zhaojun Zhang, Fangang Meng, Xiaoye Zhang, Zheng Zhou, Baoxiang Li, Fan Li, Lixiang Liu, Hongmei Shen

**Affiliations:** ^1^ Centre for Endemic Disease Control, Chinese Centre for Disease Control and Prevention, Harbin Medical University, Harbin City, China; ^2^ College of Medical Laboratory Science and Technology, Harbin Medical University (Daqing), Daqing, China; ^3^ National Health Commission and Education Bureau of Heilongjiang Province, Key Laboratory of Etiology and Epidemiology, Harbin Medical University, Harbin, China; ^4^ Heilongjiang Provincial Key Laboratory of Trace Elements and Human Health, Harbin Medical University, Harbin, China

**Keywords:** endemic neurological cretin, iodine-deficient area, subclinical hypothyroidism, nodules, neurological signs, thyroid function

## Abstract

**Backgroud:**

Endemic cretinism is the most severe manifestation among the iodine deficiency-related disorders. The clinical status of the cretins may be modified subsequently by the duration and severity of the disease. We aimed to reassess the clinical status and thyroid function of 31 surviving “neurological cretins” after 42 years of iodine supplementation in a historically severely iodine deficiency area of China.

**Methods:**

It was a cross-sectional study in design and we investigated all 31 surviving neurological cretins and 85 controls. A detailed neurological examination was conducted on each patients. All the participants were given a questionnaire and underwent B-mode ultrasonography of the thyroid. The serum levels of thyroid hormones, thyroid antibodies, serum iodine concentration (SIC) and urine iodine concentration (UIC) were measured.

**Results:**

The neurological cretins had shorter stature than that of the control. Neurological damage is still present in patients with cretinism. The prevalence of subclinical hypothyroidism and thyroid nodule in the cretins was significantly higher (*χ^2^ =*4.766, *P*=0.029 and *χ^2^ =*17.077, *P*<0.0001, respectively) compared with the control. After adjusting for confounding factors, endemic neurocretinism was found to be an independent risk factor for subclinical hypothyroidism (OR=4.412; 95% CI: 1.358–14.334; *P*=0.014) and thyroid nodule (OR=6.433; 95% CI: 2.323–17.816; *P*<0.0001).

**Conclusions:**

Iodine supplementation after birth does not reverse the neurological damage that results from maternal/foetal hypothyroidism *in utero* and is subsequently manifested as neurological cretinism. There is a cross-sectional association between endemic neurocretinism and subclinical hypothyroidism and thyroid nodule.

## Introduction

Iodine is an essential micronutrient for thyroid hormone synthesis (1). Thyroid hormone is particularly critical for normal fetal and infant neurodevelopment, therefore, adequate maternal iodine nutrition is essential during pregnancy (2), and severe iodine deficiency during pregnancy may result in fetal hypothyroidism as well as serious neurologic and cognitive defificits in children (3).

Endemic cretinism is the most severe manifestation among the iodine deficiency-related disorders (4). As early as 1908, two types of cretins are distinguished by McCarrison (5): neurological cretins are characterized by profound neurologic dysfunction, deaf-mutism, cerebral diplegia but clinical euthyroidism. Neurological abnormalities occurred *in utero* due to both maternal and fetal hypothyroxinemia caused by severe iodine deficiency. Postnatally, the persistence of hypothyroidism entails the development of myxedematous cretinism (6). Consequently, myxedematous cretins are traditionally characterized by stunted growth and signs and symptoms of hypothyroidism (7). Later studies have demonstrated that this overlap is greater than previously thought (8–10). In 1993, Boyages & Halpern (6) have proposed that both neurological cretins and myxedematous cretins are associated with severe iodine deficiency, with a time-dependent effect. For example, whatever the cause of thyroid destruction in myxedematous cretins, it appears not operative at birth but becomes an important factor with increasing age. Considering that the clinical status of the myxedematous cretins were subsequently modified by the duration length and severity of hypothyroidism (11, 12), we were curious that whether the neurological cretins also have thyroid function or clinical status changes with increasing age.

Therefore, we investigated all 31 surviving cretins in a historically severe iodine deficiency area, Jixian Village, Heilongjiang Province, China. Our institute had conducted a survey in this village in 1979, finding that the goitre rate was estimated to be 74% and the prevelence of cretinism was as high as 11.04% (145 people in 1313) (13, 14). Now, 42 years have passed, we surveyed this village again, the aim of our study is to evaluate the thyroid function and clinical signs of the endemic cretins after salt iodine supplementing with increasing age.

## Materials and Methods

### Survey Areas

Our study was performed in a district named Jixian Village located in the Huachuan County, Heilongjiang Province, which lies on the west side of Sanjiang Plain in the northeast of the People’s Republic of China. In this sparsely populated region with low terrain, inhabitants mainly rely on subsistence agriculture for their livelihood.

### Survey Subjects

This was a cross-sectional study in design and we have evaluated all the surviving cretins (n=31) who have been diagnosed as neurological cretins with marked intellectual disability in 1979 by expert physicians according to the diagnosis criteria of cretinism. All the 31 cretins have been living in Jixian Village since they were born. According to the inclusion and exclusion criteria, we surveyed 85 adults as the control group, who were from the same village as the cretins, with similar diet and socioeconomic status. The inclusion criteria were as follows: age ranges from 43 to 79 (the same age range as the cretins); and resided locally since they were born. The exclusion criteria were as follows: pregnant or lactating women; those with no blood or urine samples for determination or with missing information on key sociodemographic and lifestyle characteristics; Individuals taking medications (amiodarone etal.) that interfere with thyroid function; participants with family history of cretinism.

The project was approved by the Ethics Review Committee of Harbin Medical University (No. hrbmuecdc20201201). Participants in the control group signed informed consent before data collection and the families or guardians of the cretins also consented to participate in the study voluntarily.

### Survey Methods

Detailed neurologic examination of each cretin patient was performed by two expert physicians.

#### Intelligence Testing

Subjects were given the Raven’s Progressive Matrices (RPM) for intelligence tests. The RPM is regarded as the most well researched of all the nonverbal measures (15). It is most valuable for use with people whose test performance may be confounded by language, hearing, or motor impairments or those who are non-English speaking (16). The RPM is the recommended method of intelligence test for diagnosing cretinism in China, and the type of intelligence disability was classified as severe for IQ <25, moderate for 25-39 and mild for 40-54.

#### Audiometry

Audiometric assessment was carried out on 29 patients, the mean hearing threshold (dB) is given as the average of the hearing thresholds at 500Hz, 1000Hz and 2000 Hz (17).

A neurological examination was undertaken, and signs of paralysis and motor spasticity were particularly sought. Special attention was focused on the gait.

We have performed several physical examinations for all the participants in this study, variables were height; weight; body mass index (BMI; height/weight^2^ [kg/m^2^]) (18). A standard questionnaire was designed to acquire demographic characteristics.

#### Laboratory Test and Clinical Diagnosis

5 samples in total from east, south, west, north, and central locations were collected from the Jixian Village. Each water sample was at least 15 ml. Water samples were stored at 4°C until analysis. The As^3+^-Ce^4+^ catalytic spectrophotometry method was used for the determination of the iodine concentration of drinking water (19).

Salt samples were collected from all of the participants. Each participant provided at least 50g of household use of table salt in a clean, labeled ziplock bag. The iodine content in the salt samples was determined using the general test method of the salt industry (20). The standard salt iodine content in the village of our study was 25 mg/kg ( ± 30%) (21). From each participant, a single spot urine sample was collected in the morning in clean plastic tubes and stored at 4°C, and measured within four months of collection. UIC was measured according to the standard procedure method for determination of iodine in urine by As^3+^-Ce^4+^ catalytic spectrophotometry (22). The reference values of iodine deficiency in adults was as a median urinary iodine (MUI) <100µg/L, iodine adequate 100–299 µg/L, iodine excess ≥300 µg/L (23, 24).

Venous blood samples were collected from each subject after fasting for 8h. The supernatant was centrifuged and stored in a low-temperature refrigerator at −80°C until analysis to measure serum iodine concentration (SIC) and thyroid function. SIC was measured using the As3+-Ce4+ catalytic spectrophotometry method (25). The reference range of SIC in general population determined by the World Health Organization (WHO) is 45–90 µg/L (26).

The serum levels of FT_3_, FT_4_, TSH, Tg, TPOAb, TGAb were determined through electrochemiluminescent immunoassays using a Cobas Elesys 601 instrument (Roche Diagnostics Ltd., Switzerland). The reference values were 3.1–6.8 pmol/L FT_3_, 12–22pmol/L FT_4_, 0.27–4.2 mIU/L TSH, 0–34 IU/ml TPOAb, 0–115 IU/ml TgAb, and 3.5–77 ng/ml Tg (obtained from the manufacturer). The diagnostic criteria for thyroid disease were as follows (27): hypothyroxinaemia, FT_4_ < 12 pmol/l and TSH within the normal range; overt hypothyroidism, TSH > 4.20 mIU/L and FT_4_ < 12 pmol/l; subclinical hypothyroidism, TSH > 4.20 mIU/L and FT_4_ within the normal range; overt hyperthyroidism, TSH < 0.27 mIU/L, FT_4_ > 22 pmol/l, and FT_3_ > 6.8 pmol/l; subclinical hyperthyroidism, TSH < 0.27 mIU/L, and FT_3_ and FT_4_ within the normal range.

In addition, all participants underwent thyroid ultrasonography by experienced radiologists, using a portable instrument (LOGIQ 100 PRO, GE, Milwaukee, WI, USA with 7.5 MHz linear transducers). Subjects were examined in a sitting position with the neck hyperextended to fully expose the thyroid (28). Thyroid volume was calculated with the following formula: V (mL) = 0.479×d (mm)×w (mm)×l (mm)×0.001 (29). The diagnostic criteria for goiter, thyroid volume was > 25 ml (male) and > 18 ml (female) (30, 31); and thyroid nodule, one or more nodule (> 5mm) without goiter (27).

### Statistical Analysis

SPSS V.23.0 was used for statistical analysis. Normally distributed variables were expressed as a mean and standard deviation $\left(\bar{x}\pm \;s\right)$ , and the difference between the cretins and control were compared using the independent-samples *t*-test. Non-normally distributed variables were expressed as a median and inter-quartile range, and the *Mann-Whitney U* test was performed to compare the difference between the groups. Categorical variables and ordinal variables were expressed as a number (%), and were compared the difference between the cretins and control using the *χ^2^* test and the *Mann-Whitney U* test, respectively. Logistic regression was performed to analyze the risk factors associated with the subclinical hypothyroidism and thyroid nodule. With versus without the subclinical hypothyroidism and thyroid nodule were taken as dependent variables. The stepwise method was used to filter independent variables. The test level of alpha was set at 0.05 (two-sided), and *P*<0.05 was considered statistically significant.

## Results

In this study, the median water iodine concentration in Jixian Village was 4.5μg/L. A median water iodine concentration ≤ 10 μg/L is defined as an iodine deficiency area according to the Chinese national standard (32). The iodine content of salt samples was 26.76 ± 3.24mg/kg. In addition, the coverage of household use of qualified iodized salt in this village was 98.3%, which is in agreement with the evaluation content and criteria for the elimination of iodine deficiency diseases in China (coverage of household use of qualified iodized salt > 90.0%) (33).

### Demographic Characteristics and Clinical Status for the Neurological Cretin Patients

The basic demographic characteristics and clinical signs of the cretins were described in **Table 1**, including degree of intellectual disability, deafmutism, neurological signs, facial features, thyroid ultrasonography and individual TSH levels. In addition, the IQ level of the local control population was 88 (79~95).

**Table 1:** Clinial findings in the endemic neurological cretins.

Patient	Sex	Age	Height	Intellectual disability	Deafmutism	Neurological signs	Facial features	thyroid ultrasonography		TSH
No.	F/M	(yr)	(cm)	+Slight	+Deafness mutism/ ± Moderate impaired/Normal	Paralysis▲/Motor spasticity and Gait disorder #	A:Laugh stupidly	Thyroid Volumes (GoiterØ)	Thyroid nodule※	mIU/L (0.27–4.2)
				++Moderate			B:ocular hypertelorism			
				+++Severe			C:flat nose			
							D:salivation			
1	M	73	162	+++	^1^NA	▲	A B C D	10.57	_	2.21
2	M	44	160	+++	+	#	B C	14.13	_	3.95
3	M	51	176	+	+	#	B C	5.65	_	4.53
4	M	71	156	+++	^1^NA	▲	A B C	7.74	※	4.80
5	M	66	164	+	Normal	#	Normal	6.13	_	0.96
6	M	68	165	++	±	#	A B	9.49	※	5.68
7	M	65	169	+	±	#	B C	14.13	_	2.75
8	M	62	156	++	±	#	B C D	18.84	※	5.60
9	M	43	162	+	Normal	#	B C	5.66	_	3.35
10	M	46	166	++	±	#	A B D	9.34	_	3.10
11	M	71	163	++	±	#	B	7.86	※	20.97
12	M	67	153	++	Normal	#	B C	8.92	※	25.44
13	M	57	165	++	+	#	A B	8.63	※	3.45
14	M	64	164	++	±	#	B C	8.39	※	1.42
15	M	48	158	++	±	#	B C	4.93	※	3.15
16	M	48	166	+	±	#	B C	5.06	_	2.47
17	M	43	169	+	Normal	#	B C	4.93	※	0.69
Total (M)	M=17	58.06 ± 11.04	163.18 ± 5.60	+ = 6 ++ = 8 +++ = 3	NA=2 +=3 ±=8 Normal=4	▲ = 2 # = 15	A=5 B=16 C=12 D=3 Normal=1	8.85 ± 3.83	※=9 - =8	5.56 ± 6.84
1	F	54	155	+	±	#	BC	5.13	_	4.59
2	F	61	145	+++	+	#	A B C	6.32	※	2.57
3	F	58	156	++	±	#	B C	8.05	_	1.52
4	F	61	154	++	Normal	#	B C	12.50	※	1.08
5	F	50	160	++	+	#	B C	7.00	_	4.28
6	F	74	145	+	±	#	B C	(Ø)	※	1.15
7	F	58	155	+	+	#	B C	9.31	_	1.15
8	F	56	151	++	±	#	B C	8.40	_	5.10
9	F	60	141	+	+	#	A B C D	6.99	※	30.66
10	F	70	144	++	+	#	A B C	8.28	※	0.70
11	F	71	157	+++	Normal	#	A B C	15.47	_	0.75
12	F	64	159	+	±	#	A B C	10.37	※	0.10
13	F	70	148	+	±	#	B C	15.03	※	3.70
14	F	51	145	+++	+	#	A B C	10.10	※	5.92
Total (F)	F=14	61.29 ± 7.61	151.07 ± 6.28	+ = 6 ++ = 5 +++ = 3	+=6 ±=6 Normal=2	#=14	A=6 B=14 C=14 D=1	9.46 ± 3.20	※=8 - =6	4.52 ± 7.76
Total	31	59.52 ± 9.63	157.71 ± 8.45	+ = 12 ++ = 13 +++ = 6	NA=2 +=9 ±=14 Normal=6 ^1^NA: Not Assessed	▲ = 2 # = 29	A=11 B=30 C=26 D=4 Normal=1	9.11 ± 3.52	※=17 - =14	5.09 ± 7.16

Intellectual disability: +Mild (IQ, 40-54); ++Moderate (IQ, 25-39); +++Severe (IQ <25).

Ø: Goiter, beyond the measuring range of the instrument.

**Figure 1:**
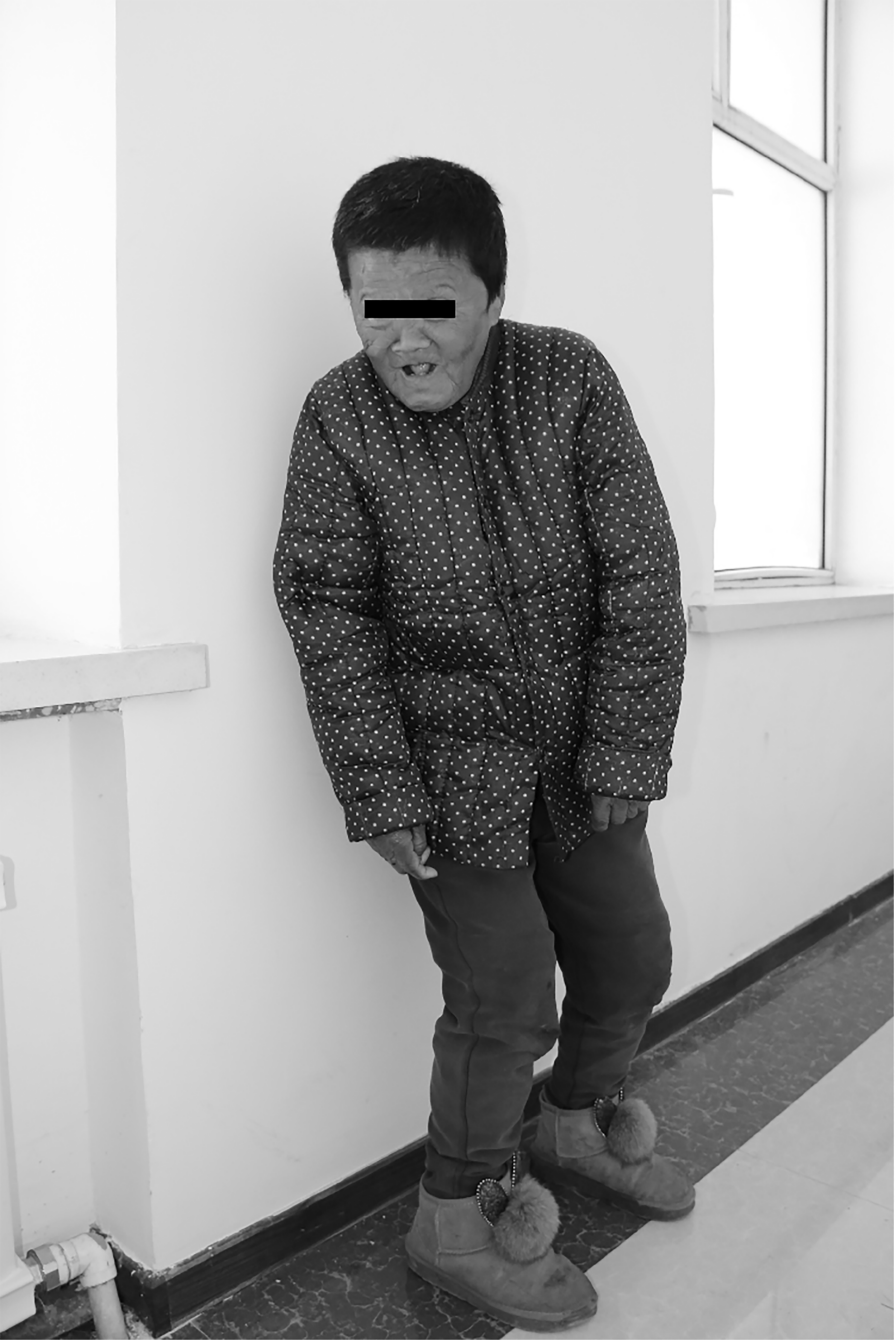
An endemic neurological cretin patient (Female, 60 years old, 141 cm, 45.9 kg). Characterized by severe intellectual impairment, deaf mutism and motor spasticity. Showing characteristic postural abnormality of endemic neurological cretin cretinism. There is knock knee, but stance is moderately wide-based. The arms are held with the shoulders abducted, and the elbows flexed. Huachuan, Heilongjiang Province, PRC, 2020.

Of the 31 neurological cretins, 17 were male and 14 were female. The average age was 59.52 ± 9.63 years. 27 of 31 neurological cretins with ataxic gait, with marked motor spasticity, walked with bent knees, and their arms tended to be held in flexion (**Figure 1**). In 2 patients, standing up was impossible.

Audiologic examination revealed deafmutism or moderate neurologic hypoacusia in 23 of the 29 subjects examined, while for the remaining 2 patients, the audiometry was unobtainable because of the presence of defective attention or severe intellectual disability. It is worth mentioning that, 6 were deemed to have no hearing or speech difficulties. Most of the patients, to some extent, had typical cretinism facial features, such as laughing stupidly, ocular hypertelorism, flat nose or salivation. We did not find similar physical neurological abnormalities in the control group.

### Basic Demographic Characteristics in the Neurological Cretin Patients and Control

Most of the neurological cretins, whether males or females, had short stature, and the average height of the male cretins (163.18 ± 5.60) was significantly lower (p<0.05) than that of the control group (167.70 ± 5.45) living in the same endemic area; and of the female cretins the average height (151.07 ± 6.28) was significantly lower (p<0.01) than that of the control group (156.44 ± 6.26) (**Table 2**).

**Table 2:** Demographic characteristics in the neurological cretins and control.

Characteristics	Total	Endemic cretins (*n*=31)	Control (*n*=85)	*P* Value
Gender *n* (%)				
Male	47 (40.5)	17 (54.8)	30 (35.3)	0.058
Female	69 (59.5)	14 (45.2)	55 (64.7)	
Age(years) *n* (%)				
41–50	31 (26.7)	7 (22.6)	24 (28.2)	0.127
51–60	42 (36.2)	8 (25.8)	34 (40.0)	
61–70	29 (25.0)	11(35.5)	18 (21.2)	
71–79	14 (12.1)	5 (16.1)	9 (10.6)	
Height (cm) $\bar{x}\pm s$				
Total	159.62 ± 8.21	157.71 ± 8.45	160.39 ± 8.05	0.126
Male	165.95 ± 5.89	163.18 ± 5.60	167.70 ± 5.45	0.013^*^
Female	155.27 ± 6.61	151.07 ± 6.28	156.44 ± 6.26	0.006^*^
*P* Value	<0.0001*	<0.0001*	<0.0001*	
Weight (kg) $\bar{x}\pm s$				
Total	61.95 ± 11.08	59.01 ± 10.92	63.13 ± 10.99	0.080
Male	66.08 ± 11.41	61.72 ± 9.60	68.82 ± 11.77	0.043^*^
Female	59.11 ± 9.98	55.71 ± 11.86	60.06 ± 9.30	0.151
*P* Value	0.001^*^	0.130	0.001^*^	
BMI (kg/m^2^) $\bar{x}\pm s$				
Total	24.25 ± 3.53	23.69 ± 3.74	24.47 ± 3.44	0.299
Male	23.92 ± 3.46	23.15 ± 3.02	24.41 ± 3.68	0.245
Female	24.47 ± 3.58	24.35 ± 4.50	24.51 ± 3.33	0.882
*P* Value	0.427	0.384	0.903	

BMI, boy mass index; A t-test was used for height, weight and BMI; the Mann-Whitney U test was adopted for age; a χ^2^ test was used for gender.

^*^P < 0.05 and the difference between groups was statistically significant.

### Iodine Nutrition Status and Thyroid Function in the Neurological Cretin Patients and Control

The UIC, SIC and thyroid function in the neurological cretins and control are shown in **Table 3**. There were no statistically significant differences in both the median UIC (168.92 Vs 175.86μg/L) and the median SIC levels (62.39 Vs 63.82μg/L) between the neurological cretins and the control.

**Table 3:** The iodine status and thyroid function in the neurological cretins and control.

Characteristics	Total	Endemic cretins (*n*=31)	Control (*n*=85)	*P* Value
UIC (μg/l) [M, Q]	169.90 (123.65,243.89)	168.92 (123.95,202.39)	175.86 (123.65,263.35)	0.378
SIC (μg/l) [M, Q]	63.60 (54.03,71.69)	62.39 (54.63,71.70)	63.82 (53.85,71.68)	0.830
FT_3_ (pmol/l) [M, Q]	5.40 (4.95,5.82)	5.60 (5.34,5.90)	5.29 (4.89,5.80)	0.070
FT_4_ (pmol/l) [M,Q]	16.27 (14.97,18.30)	15.94 (14.96,18.09)	16.39 (14.92,18.57)	0.518
TSH (µIU/ml) [M, Q]	2.33 (1.26,3.67)	3.35 (1.42,5.10)	2.06 (1.24,3.25)	0.009^*^
Tg (ng/ml) [M, Q]	11.84 (5.57,34.05)	23.97 (7.77,58.39)	10.68 (5.07,25.53)	0.060
TPOAb (+), *n* (%)	13 (11.2)	5 (16.1)	8 (9.4)	0.495
TgAb (+), *n* (%)	12 (10.3)	5 (16.1)	7 (8.2)	0.373

[M, Q], [median, inter-quartile range]; UIC, urinary iodine concentration; SIC, serum iodine concentration; FT_3_, free triiodothyronine; FT_4_, free thyroxine; TSH, thyroid stimulating hormone; Tg, thyroglobulin; TPOAb (+), thyroid peroxidase antibody-positive; TgAb (+), thyroglobulin antibody-positive.

Mann-Whitney U test was adopted for UIC, SIC, FT_3_, FT_4_, TSH, Tg; χ^2^ test was used for TPOAb (+) and TgAb (+). The normal reference ranges were obtained from the manufacturer: FT3, 3.10-6.80 pmol/L; FT4, 12.00-22.00 pmol/L; TSH, 0.27-4.20 mIU/L; TPOAb, 0-34 IU/mL; TgAb, 0-115 IU/mL; Tg, 3.5–77 ng/ml.

^*^P < 0.05 was considered significant.

Compared with the control group, the TSH levels of the neurological cretins were significantly higher (3.35 µIU/ml vs. 2.06µIU/ml, P<0.01). No statistical difference was detected in median FT_3_ concentrations, median FT_4_ concentrations, and TgAb and TPOAb-positive rates between the neurological cretins and control. There were no differences in Tg levels between the neurological cretins and control.

The prevalence of subclinical hypothyroidism and thyroid nodule in the neurological cretins was significantly higher (*P* = 0.029 and *P* < 0.0001, respectively) compared with the control. Nevertheless, we observed that there was no significant difference in the prevalence of other thyroid diseases between the two groups (**Table 4**).

**Table 4:** Prevalence of thyroid disease between neurological cretins and control (Number of thyroid disease and percentages).

	Endemic cretins (N=31)	Control (N=85)	*P* Value
	*n* (%)	*n* (%)	
Hypothyroxinaemia			
Yes	0 (0.0)	1 (1.2)	1.000
No	31 (100.0)	84 (98.8)	
Overt hypothyroidism			
Yes	3 (9.7)	1 (1.2)	0.100
No	28 (90.3)	84 (98.8)	
Subclinical hypothyroidism			
Yes	8 (25.8)	7 (8.2)	0.029^*^
No	23 (74.2)	78 (91.8)	
Overt hyperthyroidism			
Yes	1 (3.2)	2 (2.4)	1.000
No	30 (96.8)	83 (97.6)	
Subclinical hyperthyroidism			
Yes	0 (0.0)	1 (1.2)	1.000
No	31 (100.0)	84 (98.8)	
Autoimmune thyroid disease			
Yes	7 (22.6)	10 (11.8)	0.246
No	24 (77.4)	75 (88.2)	
Thyroid nodule			
Yes	17 (54.8)	14 (16.5)	<0.0001^*^
No	14 (45.2)	71 (83.5)	
Goiter			
Yes	1 (3.2)	3 (3.5)	0.720
No	30 (96.8)	82 (96.5)	

^*^P < 0.05 and the difference between groups was statistically significant.

### Logistic Regression Analysis Between Neurocretinism and Subclinical Hypothyroidism and Thyroid Nodule

Because of the high prevalence of subclinical hypothyroidism and thyroid nodule in neurocretinism in single-factor analysis. To further determine whether the prevalences of subclinical hypothyroidism and thyroid nodule were associated with endemic neurocretinism in multivariate analysis, binary logistic regression models were used. After adjusting for confounding factors, endemic neurocretinism was found to be an independent risk factor for subclinical hypothyroidism (OR=4.412; 95% CI: 1.358–14.334; P=0.014) and thyroid nodule (OR=6.433; 95% CI: 2.323–17.816; P<0.0001). Refer to **Table 5** for details.

**Table 5:** Risk factors associated with subclinical hypothyroidism and thyroid nodule.

	Model	*β*	*S_x_*	Wald *χ* ^2^	OR (95%CI)	*P* Value
Subclinical hypothyroidism						
Endemic cretinism						
No	Ref					
Yes	1	2.123	0.757	7.864	8.353 (1.895, 36.823)	0.005
	2	1.484	0.601	6.097	4.412 (1.358, 14.334)	0.014
Thyroid nodule						
Endemic cretinism						
No	Ref					
Yes	1	2.056	0.557	13.637	7.812 (2.624, 23.260)	<0.0001
	2	1.861	0.520	12.825	6.433 (2.323, 17.816)	<0.0001

Binary logistic regression analysis was performed to estimate odds ratio for endemic cretinism for subjects in two models: model 1 (unadjusted) and model 2 (adjusted for age, gender).

OR, odds ratio; 95% CI, 95% confidence interval.

## Discussion

The Jixian Village, as a typical area we selected to investigate is once a severe iodine deficiency area. Due to a large number of cretinis, Jixian Village was once called “Village of Fools” and thus became famous throughout China in the last century. Now it is one of a few villages with so many existing endemic cretin patients for study in China. All the 31 cretins were born before the introduction of iodine prophylaxis, the IQ level of the local control population was 88 (79~95) which matched the moderate IQ level according to the national Chinese standards. Consequently, it is admirable that the introduction of iodized salt into Jixian village in 1978 prevented the occurrence of any further neurological cretinism from that date onwards. Since the investigation in 1979 of endemic goiter and endemic cretinism, all patients diagnosed with endemic goiter have been treated with oral potassium iodide. The thyroid goiter rate decreased from 74% in 1979 to 22.38% in 1982, according to a census taken three years later (34). During the second census conducted in the same area in 2002, the rate of thyroid goiter declined from 74% to 18.6% (35). In this survey, the thyroid goiter rate in cretin patients and control population was 3.2% and 3.5%, respectively. According to a nationally representative cross-sectional study with 78,470 participants from all 31 provincial regions of China, the weighted prevalence of goiter in adults was 1.17% (27). By comparison, the thyroid goiter rate in Jixian is the same as other regions, which indicates that the long-term mandatory USI program is effective in preventing iodine deficiency disorders.

UIC is a reliable indicator for the iodine status assessment because 90% of dietary iodine is excreted in urine within 24 hours after consumption (36). Due to the sensitivity of serum iodine in evaluating individual iodine nutrition status, the stability of population iodine nutrition status, and the significance of screening in people at high risk for thyroid disease (37), our study included SIC as well as the UIC for the iodine status assessment. According to the recommendations of the WHO/UNICEF/ICCIDD (24, 26, 38), whether from the perspective of the median UIC or the median SIC, the population iodine status was adequate both in the cretins and the control, which indicates that the policy of salt iodization is well implemented and successful in this area. However, it is worth noting that no statistically significant differences were found in both the median UIC and SIC levels between the neurological cretins and the control. The result shows that there is no abnormal iodine metabolism in neurological cretins after salt iodization, compared with control.

The neurological cretins, male or female, had shorter stature than that of the control group, which indicated that with increasing age, the height of neurocretinisms may also be affected. However this finding is not consistent with previously published data which have shown that growth retardation was found at a much greater frequency in myxedematous cretins (15, 39–41), rather than in neurological cretins, while for neurological cretins, they may have postnatal growth retardation, but may eventually be normal or approximately normal when they be adults (42). It seems that there is no reversal for the deaf-mutism and neurological damage for these neurological cretins even after receiving iodine supplement. This reinforces the conclusion of previous publications that treatment after delivery may improve brain growth and developmental achievement slightly, but it does not reverse the neurological damage that results *in utero* from maternal/foetal hypothyroidism and is subsequently manifested as neurological cretinism (43). The surviving neurological cretins are more in signs of mild intellectual disability and neurological signs, and they can still participate in family activities to some extent. The reason for this variability in signs of neurologic involvement among different surveys may be explained by the age differences of the subjects or by the different methods used to detect neurologic and audiological deficits (44). Furthermore, researches have shown that the variability of neurologic clinical signs among the types of cretinism may be explained both by environmental factors and by the severity and duration of fetal and postnatal hypothyroidism, which could modify the phenotypic expression of the disorder (9, 11).

In this study, high prevalence of subclinical hypothyroidism and thyroid nodule was found in neurological cretin patients. Although the cross-sectional study which we used makes it difficult to determine the causal relationship between the neurological cretins and subclinical hypothyroidism and thyroid nodule, the historical data from previous investigation of the village shows that all cretins were clinical euthyroidism at the time of diagnosis in 1979 (FT_4_: 9.83 ± 1.91μg/dl; TSH: 6.14 ± 3.49mIU/L, measured by radioimmunoassay with a reference value of 0-10 mIU/L) (13). Although endemic neurological cretins are caused by maternal/fetal hypothyroidism *in utero*, and they may have only transient hypothyroidism in the postnatal period, we speculate these cretin patients are more likely to be affected by iodine deficiency or other mechanisms affecting thyroid development. Also, they are more prone to thyroid dysfunction and morphological abnormalities in the future, such as subclinical hypothyroidism and thyroid nodules in our study. Biondi B point that subclinical hypothyroidism is common and most individuals can be observed without treatment, also there is no evidence that levothyroxine therapy is beneficial to persons aged 65 or older (45). Nevertheless, the endemic neurocretinism patients also should be followed up regularly to prevent the development of overt hypothyroidism.

In our study, the prevalence of thyroid nodules in the neurological cretins (54.8%) was much higher than normal, while the condition of the control group (16.5%) was similar to that of the normal populations. Numerous studies suggest a prevalence of 19-35% with ultrasound data (46). According to the epidemiological evidence from 31 provinces of mainland China, the weighted prevalence of thyroid nodules in adults was 20.43%, and iodine deficiency was significantly associated with higher odds of most thyroid disorders (27). Gharib also reported that iodine deficiency seem to increase the risk of thyroid nodules (47). The high prevalence of nodules in the cretins did still so high even after iodine supplementation, because studies show that the thyroid gland before birth and in the first few years of life (<4 years old) is able to respond physiologically to iodine deficiency and supplementation, while in patients with endemic cretinism there is a reduction in response to iodine supplementation with increasing age (11, 12, 48). Fortunately, very few of these lesions ultimately prove to be malignant (about 5%) (49). Nonetheless, as recognized risk factors for thyroid malignancy, nodules that are firm, fixed, or rapidly growing require prompt evaluation (50). It also requires more regular follow-up checks for the existing endemic neurocretinism patients.

This study had some limitations. First, the survey was confined to a unique village, although 31 cretins were all cases we investigated as hard as we could, while the results might not be representative because of the relatively small sample size, which may affect the authenticity of the results. It is expected that this problem can be solved by a larger sample or multi-center validation in other areas. Second, survivor bias may exist due to the since most of the 31 existing cretin patients surveyed had mild clinical status because of the death of patients with severe conditions.

## Conclusion

In conclusion, in this historically severe iodine-deficient area, under the implementation of a comprehensive salt iodization policy, endemic neurological cretins had normal iodine nutrition. The institution of iodine supplementation after birth does not reverse the neurological damage that results *in utero* from maternal/foetal hypothyroidism and is subsequently manifested as neurological cretinism. We found a cross-sectional association between endemic neurological cretins and subclinical hypothyroidism and thyroid nodule.

## Data Availability Statement

The original contributions presented in the study are included in the article/supplementary material. Further inquiries can be directed to the corresponding authors.

## Ethics Statement

The project was approved by the Ethics Review Committee of Harbin Medical University (No. hrbmuecdc20201201). Harbin Medical University is the authors’ affiliation. The patients/participants provided their written informed consent to participate in this study. Written informed consent was obtained from the individual(s) for the publication of any potentially identifiable images or data included in this article.

## Author Contributions

HS, designed the study. LL, project administration. JL, data curation and writing- original draft. HS, LL, ZZha, FM, XZ, JL, YH, BR, ZZho, BL, FL, investigation. All authors revised the report and approved the final version before submission.

## Funding

This work was supported by the National Natural Science Foundation of China (grant no. 82073490) and the Fundamental Research Funds for the Provincial Universities (grant no. JFXN201909).

## Conflict of Interest

The authors declare that the research was conducted in the absence of any commercial or financial relationships that could be construed as a potential conflict of interest.

## Publisher’s Note

All claims expressed in this article are solely those of the authors and do not necessarily represent those of their affiliated organizations, or those of the publisher, the editors and the reviewers. Any product that may be evaluated in this article, or claim that may be made by its manufacturer, is not guaranteed or endorsed by the publisher.
